# Genetic evaluation of five patients with ROHHAD-NET using whole genome sequencing and optical genome mapping

**DOI:** 10.1186/s13023-025-03938-3

**Published:** 2025-08-07

**Authors:** N. van Engelen, H. M. van Santen, F. van Dijk, M. M. Kleisman, J. H.M. Merks, A. Y.N. Schouten-van Meeteren, E. J. Kamping, K. Neveling, A. Hoischen, M. C.J. Jongmans, R. P. Kuiper

**Affiliations:** 1 Princess Máxima Center for Pediatric Oncology, Utrecht, The Netherlands; 2 Department of Pediatric Endocrinology, Wilhelmina Children’s Hospital, Utrecht, The Netherlands; 3 Division of Imaging and Oncology, University Medical Center Utrecht, Utrecht University, Utrecht, The Netherlands; 4 Department of Human Genetics, Radboud University Medical Center, Nijmegen, The Netherlands; 5 Department of Internal Medicine, Radboud Center for Infectious Diseases (RCI), Radboud Institute for Medical Innovation Radboud University Medical Center, Nijmegen, the Netherlands; 6 Department of Genetics, University Medical Center Utrecht, Utrecht, The Netherlands

**Keywords:** ROHHAD-NET, Whole genome sequencing, Optical genome mapping, Germline

## Abstract

**Background:**

Rapid-onset obesity, hypothalamic dysfunction, hypoventilation, autonomic dysregulation (ROHHAD) and neuroendocrine tumor (NET) is a very rare condition with an unknown etiology. While various potential causes have been hypothesized, including genetic and paraneoplastic autoimmune mechanisms, no definitive cause has been identified to date. This study aimed to explore whether patients with ROHHAD-NET share an underlying heritable genetic etiology.

**Results:**

We identified five female patients clinically suspected of having ROHHAD(-NET); among them in two patients a NET was found: a ganglioneuroma and a low grade cerebellar ganglion cell tumor with BRAF mutation. To identify potential pathogenic germline genomic variants, whole genome sequencing (WGS) was performed on germline DNA from all five patients, including four patient-parent trios. Furthermore, optical genome mapping (OGM) was performed for two patients to detect germline structural variants (SVs). Rare single nucleotide variants (SNVs) and small insertions/deletions (InDels) were identified through WGS and rare SVs affecting (non)-coding or regulatory regions were analyzed using both WGS and OGM. We explored a *de novo*, inherited autosomal dominant and autosomal recessive inheritance scenario. However, no candidate variants in a recurrently affected gene locus or genomic region were identified in two or more patients.

**Conclusion:**

Our comprehensive genome-wide data analysis did not reveal evidence of a monogenetic cause for ROHHAD-NET. Whereas these findings do not exclude a genetic etiology for ROHHAD-NET, they strengthen the hypothesis of an autoimmune origin for symptoms of ROHHAD.

**Supplementary Information:**

The online version contains supplementary material available at 10.1186/s13023-025-03938-3.

## Background

ROHHAD-NET is an acronym for rapid-onset obesity, hypothalamic dysfunction, hypoventilation, autonomic dysregulation (ROHHAD), and neuroendocrine tumor (NET). This rare disease was initially described by Fishman et al. in 1965 and renamed ROHHAD in 2007 by Ize-Ludlow et al. [1, 2]. The first clinical presentation is rapid weight gain in a previously healthy child, with a median onset age of 3 years [3]. This is followed by features of hypothalamic dysfunction (growth hormone deficiency, central hypothyroidism, AVP deficiency, central adrenal insufficiency, pubertal disturbances, and hyperprolactinemia), and autonomic dysregulation (central hypoventilation, thermal dysregulation, cardiovascular manifestations, ophthalmic abnormalities, altered pain perception, gastrointestinal dysmotility) [3]. Approximately 40–56% of the ROHHAD(-NET) patients are diagnosed with a neuroendocrine tumor, including ganglioneuroma, ganglioneuroblastoma and neuroblastoma [3, 4]. The majority of the tumors are asymptomatic ganglioneuromas located at one of the adrenal glands. The tumors are diagnosed at a median age of 4.75 years, often within two years of the initial onset of obesity [3]. Additional clinical features, though less common, include seizures, developmental delay, behavioral disorders, and recurrent respiratory infection.

Currently, ROHHAD(-NET) is a clinical diagnosis because the exact etiology is unknown, therefore, no genetic test or biochemical marker is available. Various potential etiologies have been proposed including genetic, epigenetic and paraneoplastic autoimmune mechanisms. To investigate the genetic hypothesis, previous studies have performed panel-based genetic testing, whole exome sequencing (WES) and whole genome sequencing (WGS) [2, 4–14]. The genetic panels used in these studies primarily targeted genes associated with syndromes involving obesity and/or hypoventilation, e.g. *PHOX2B*, associated with congenital central hypoventilation syndrome (OMIM#209880); *RAI1*, associated with Smith-Magenis syndrome (OMIM#182290); *HTR1A*, *OTP* and *PACAP*, genes important for the development of the hypothalamus and autonomic nerve system. Several studies performed WES-based analysis on samples from approximately fourty patients suspected of ROHHAD(-NET) including germline patient-parent trio-WES analysis and tumor analysis [4, 9, 11–13]. Notably, one study investigated a monozygotic twin pair of which only one sibling exhibited a ROHHAD(-NET) phenotype [9]. Recently, Grossi et al. conducted WGS for germline DNA from two patient-parent trios [14]. Thus far, none of these studies identified a genetic variant causing ROHHAD(-NET).

In this report, we provide a clinical overview of five patients suspected of having ROHHAD(-NET), and present a comprehensive genome-wide analysis of these patients that enables detection of aberrations that were previously missed. We performed WGS on germline DNA for all five patients, including four patient-parent trios, and conducted optical genome mapping (OGM) for two patients to explore both coding and noncoding regions, as well as genomic structural variants (SVs).

## Methods

### Patient selection

The patients were identified at the Wilhelmina Children’s Hospital and the Princess Máxima Center for Pediatric Oncology in Utrecht, The Netherlands. All patients were clinically suspected of having ROHHAD-NET based on their presentation, either with rapid onset obesity at young age or signs of hypoventilation (Table 1). In two patients a neuroendocrine tumor was found. Alternative clinical or genetic diagnoses considered, like congenital central hypoventilation syndrome, Smith-Magenis syndrome, Prader-Willi syndrome (OMIM#176270) Beckwith-Wiedemann syndrome (OMIM#130650), were excluded. Structural/congenital hypothalamic-pituitary anomalies were excluded by brain MRI.

Ethical approval for the study was not required according to the medical ethics committee of the Utrecht University Medical Center (23–175/DB). The patients and/or parents provided written informed consent for inclusion in this study and publication of their data and/or the data from their children.

### Whole genome sequencing

The KAPA Hyperplus Kit (Roche Sequencing Solution, Pleasanton, CA, USA) was used to perform library preparation using DNA isolated from whole blood samples from the patients and their parents and a tumor sample from Patient 5. WGS was performed using an S4 flow cell on a Novaseq platform (Illumina, San Diego, CA, USA). Sequencing depth was targeted at 30x for the germline samples and 90x for the tumor sample. Reads were aligned to genome build GRCh38 using BWA v0.7.13 [15].

### Germline variant calling and filtering

**Fig. 1:**
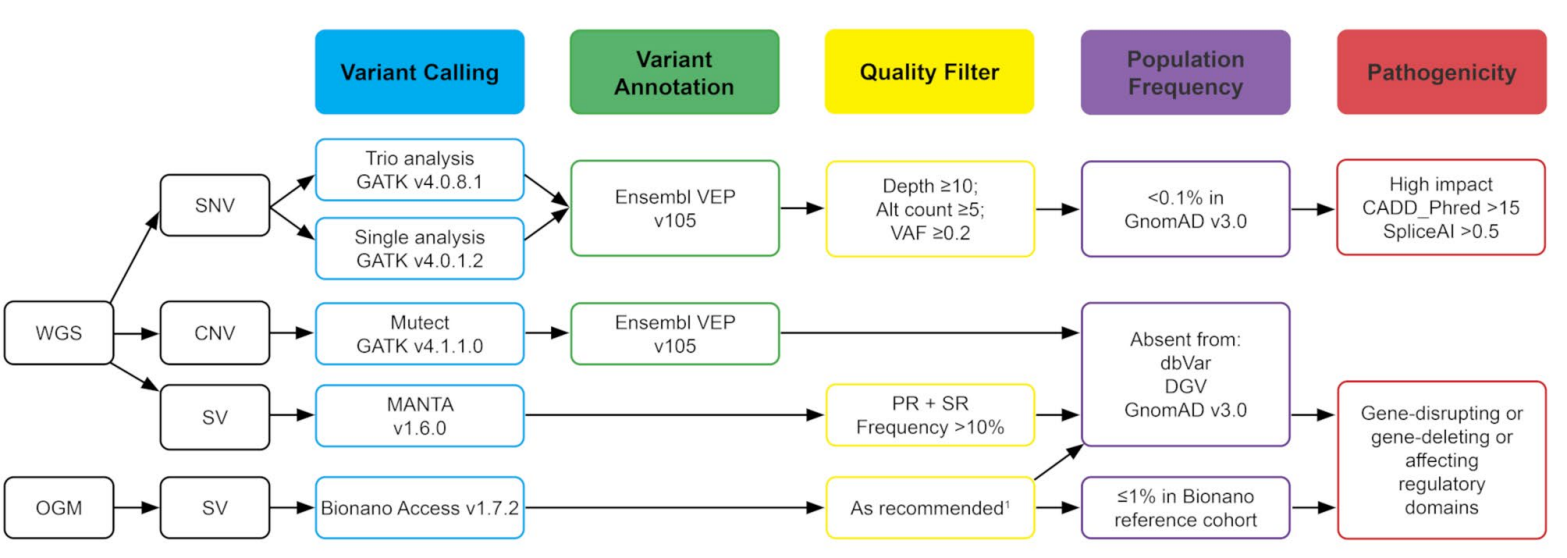
An overview of the data analysis and filtering pipeline for both germline WGS and OGM data ^1^ As previously reported by Mantere et al., 2021 [24] WGS = whole genome sequencing; OGM = optical genome mapping; SNV = single nucleotide variant; CNV = copy number variant; SV = structural variant; VAF = variant allele frequency; PR = paired read; SR = split read; DGV = Database of Genomic Variants: Structural Variation; dbVar = NCBI dbVar Curated Common Structural Variants

Calling of germline single nucleotide variants (SNVs) and small insertions and deletions (InDels) was performed using GATK best practices v4.0.1.2 [16] (Fig. 1). Variants were annotated using Ensembl Variant Effect Predictor (VEP) v105 [17], and filtered based on quality (depth ≥ 10, alt count ≥ 5, variant allele frequency (VAF) ≥ 0.2), population frequency (GnomAD v3.0 population frequency ≤ 0.1%) [18], and on the likelihood of having a pathogenic effect (High Impact consequence, a CADD_Phred score ≥ 15 and/or SpliceAI ≥ 0.5) [19, 20]. The Results section provides further details on the filtering steps applied according to the mode of inheritance.

### *De novo* variant calling and filtering

The familial concordance for each patient-parent trio dataset was validated using BAM-matcher v1.0. Variants were called for both patient and parents and *de novo* variants were detected by implementing GATK best practices v4.0.8.1 [16]. Variants were included based on quality (depth ≥ 10, alt count ≥ 5, VAF ≥ 0.2) and population frequency (GnomAD v3.0 population frequency ≤ 0.1%) [18]. All called *de novo* variants were manually validated in the Integrative Genomics Viewer (IGV) to exclude invalid calls and to confirm the ‘de novo’ status.

### Tumor variant calling and filtering

Somatic variant calling for the tumor-normal pair was performed using Mutect2 (GATK v4.1.1.0) [16]. The somatic calls were annotated using Ensembl VEP v105 [17]. SNVs, multi nucleotide variants (MNVs) and small InDels with a ‘PASS’ filter were filtered based on quality (depth ≥ 10, alt count ≥ 5, VAF ≥ 0.1) and population frequency (GnomAD v3.0 population frequency ≤ 1%) [18]. Predicted pathogenic variants were included in case of a High Impact consequence, CADD_Phred score ≥ 15 and/or SpliceAI ≥ 0.5 [19, 20].

### Copy number variant detection

Copy number variants (CNVs) were called using GATK v4.0.1.2 [16] (Fig. 1). All CNVs were manually validated in IGV using BEDgraph files. The CNVs were compared to parental data to select the most reliable *de novo* CNV calls. For Patient 5, for whom we did not have parental data, we used the data from other parents and the population databases to identify rare candidate variants. The CNVs were compared to population databases for SVs (dbVAR, DGV and GnomAD), using the UCSC browser to identify rare, *de novo* CNVs [18, 21, 22].

### Structural variant detection on WGS data

SVs were detected using MANTA v.1.6.0 [23] (Fig. 1). SVs that were called in any of the parents, based on matching start and/or end position, were excluded. Only called SVs with both paired and split read support were included based on a PR_ALT_F ≥ 0.1 and SR_ALT_F ≥ 0.1. SVs with a length of > 10.000 bp and/or with at least one of the breakpoints located in a gene were included for further evaluation. All selected SVs were visually validated in IGV and compared to the parental sequences to identify probable *de novo* SVs. For Patient 5, for whom we did not have parental data, we used the data from other parents and the population databases (dbVAR, DGV and GnomAD) to identify rare SVs [18, 21, 22].

### Optical genome mapping

For Patients 4 and 5, a whole blood sample was available to extract ultra-high molecular weight (UHMW) DNA, according to the manufacturer’s instructions (Bionano Prep SP Frozen Human Blood DNA Isolation Protocol, Bionano, San Diego, CA, USA) with small modifications as described by Mantere et al. [24]. In summary, gDNA was isolated using a nano bind disk, labeled with DL-green fluorophores and stained overnight to visualize the DNA backbone. Labeled gDNA samples were loaded on a G2.3 Saphyr chip and imaged by the Saphyr instrument (Bionano, San Diego, CA, USA). For each sample, 800 Gb of data was generated and a *de novo* assembly with subsequent comparison to the reference genome GRCh38 was performed using Bionano Access (v1.7.2) (Fig. 1). The SVs were filtered using recommended quality setting by Bionano, allowing the detection of SVs of ≥ 500 bp. Variants present in > 1% of an OGM dataset of ~ 300 human control samples were filtered out to remove common variants and artifacts. Remaining rare SVs were compared to population databases for SVs (dbVAR, DGV and GnomAD) using the UCSC browser [18, 21, 22]. SVs absent from these databases were selected for evaluation and visual validation in Bionano Access and IGV.

## Results

### Clinical information

We included five patients who were clinically suspected of having ROHHAD(-NET) without an alternative clinical or genetic diagnosis. The clinical characteristics and results of diagnostic genetic testing are summarized in Table 1. All patients were female, born to nonconsanguineous, healthy parents of Dutch ancestry, with unremarkable family histories for phenotypic features resembling ROHHAD(-NET). They presented with obesity between the age of three months and four years. Four patients were diagnosed with central hypoventilation of whom three required ventilatory support. Each patient exhibited at least one pituitary deficiency, including central hypothyroidism. Autonomic dysfunction was present in all patients, most commonly presenting as thermal instability and/or altered eye movements. Delayed motor development was observed in all patients. In two patients a tumor was detected. Patient 1 was diagnosed with a presacral ganglioneuroma at the age of 4 years, six months after the onset of obesity. In addition to tumor resection, her treatment plan also included intravenous immunoglobulin (IVIG) treatment. Patient 5 was diagnosed with a low-grade ganglion cell tumor located in the cerebellum at the age of 2 years, 1.5 years after the onset of obesity. In both patients, surgical resection was performed. In all patients, prior genetic testing, including (patient-parent) trio-WES, did not reveal an alternative diagnosis or a genetic mutation causative for ROHHAD(-NET).

### Data collection

Germline WGS data was generated for all five patients and for both parents of Patients 1–4. For two patients, OGM was performed on a germline sample (Patients 4 and 5). For Patient 5, DNA and RNA sequencing was performed on a sample from the ganglion cell tumor. An overview of the quality reports are available (Supplementary Table 1 (WGS) and 2 (OGM)).

### *De novo* model

**Fig. 2:**
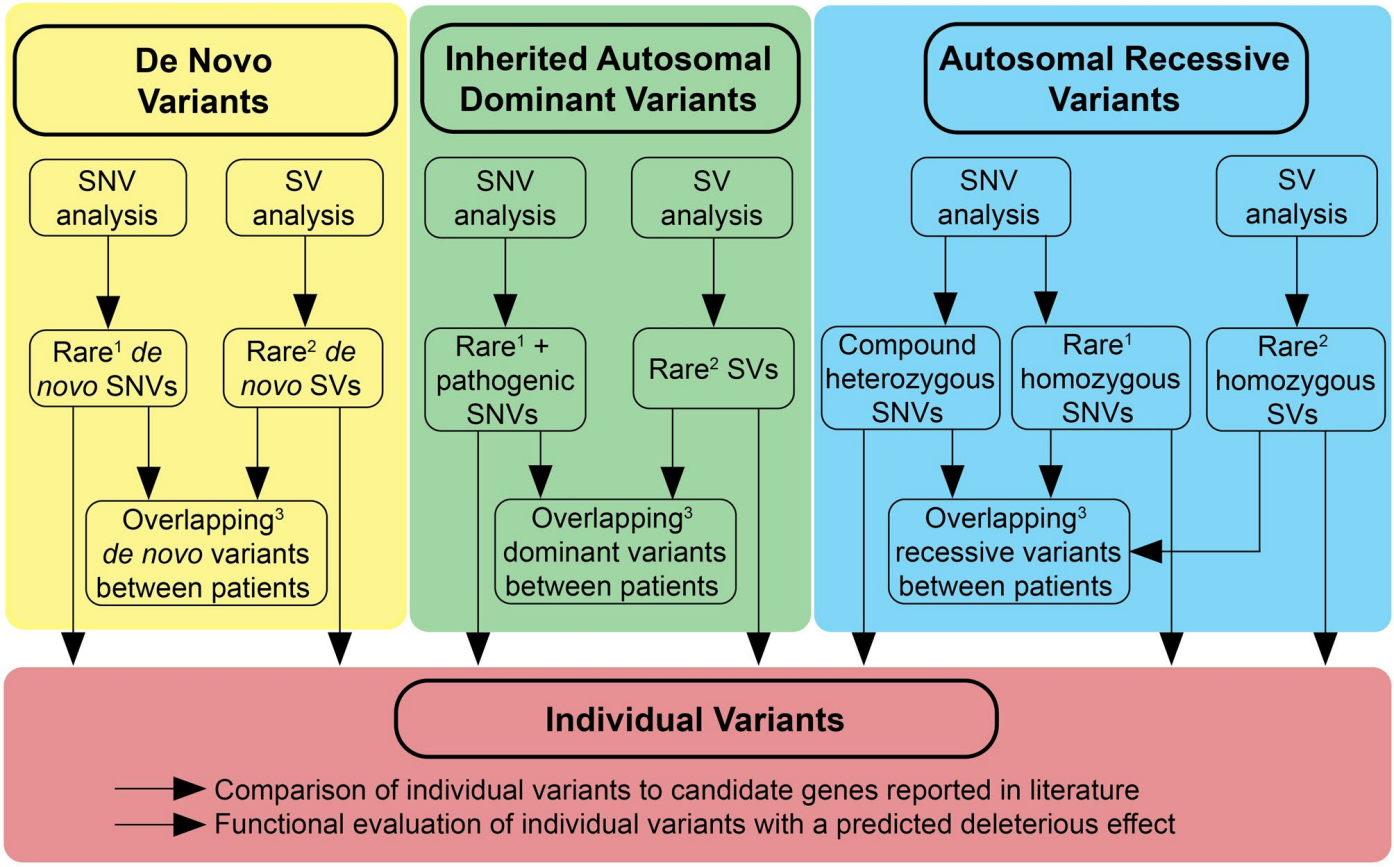
Overview of the data analysis performed for each potential scenario for a genetic ROHHAD-NET cause. De novo variants and compound heterozygous variants could only be determined for four patients for whom we have parental data SNV = single nucleotide variant; SV = structural variant. ¹ Rare SNVs = GnomAD population frequency < 0.1%; ² Rare SVs = absent from dbVAR, DGV, GnomAD; ³ Overlapping variants = based on same gene name or same region (distance < 1000 bp)

Consistent with previously reported patients, both parents and siblings of all five patients were unaffected by the condition. Given this observation and the rarity of the disease, a *de novo* pathogenic germline variant would represent the most probable scenario if ROHHAD-NET is a genetic condition. We first collected all rare *de novo* SNVs, small InDels, and SVs in patient-parent WGS data from Patients 1–4 (Fig. 2). We identified 58–107 rare possible *de novo* SNVs and InDels in each patient, including one coding *de novo* variant in Patient 4 (Supplementary Table 3). In addition, we identified three *de novo* SVs in Patients 1 and 2 (Supplementary Table 4). We explored a scenario in which a rare *de novo* variant occurred in the same gene or region (distance < 1000 bp apart) in at least two patients. Intronic *de novo* variants shared by two patients were found in one gene (*CNTNAP2)*, but these variants were not predicted to have a pathogenic effect. In addition, for the genes or regions where one patient carried a *de novo* variant, we did not identify an inherited, coding variant with a predicted pathogenic effect in a second patient.

### Autosomal dominant inheritance model

As there are no familial cases of ROHHAD-NET, an inherited autosomal dominant pathogenic variant is a less likely cause for the condition. Nevertheless, the presence of an inherited heterozygous pathogenic variant with low or incomplete penetrance cannot be excluded. Therefore, we analyzed whether rare SNVs or small InDels with a predicted pathogenic effect (CADD_Phred ≥ 20 and/or SpliceAI ≥ 0.5) identified in the five patients affected a gene or genomic region in at least two patients (Fig. 2). SVs and *de novo* variants were included in this analysis. Subsequently, variants of all recurrently affected genes or regions were analyzed (Supplementary Table 5). Based on current knowledge about the function of these genes, no true candidate gene was identified.

We had access to tumor data from Patient 5, in which we performed a second hit analysis, i.e. we analyzed whether any gene in which we identified a predicted deleterious heterozygous germline variant was affected by loss of heterozygosity or carried an additional damaging somatic aberration (Supplementary Table 10). We did not detect any clonal aberrations in the tumor meeting these criteria.

### Autosomal recessive inheritance model

To explore possible candidates for an autosomal recessive model, we identified all rare homozygous SNVs and small InDels, and coding compound heterozygous SNVs and small InDels from all five patients (Fig. 2, Supplementary Table 6). We excluded homozygous variants which were also homozygous in at least one of the patient’s parents and only included variants with a predicted pathogenic effect (CADD_Phred ≥ 20 and/or SpliceAI ≥ 0.5). We compared the biallelic SNVs and small InDels based on gene name and region between the patients and to the biallelic SVs but did not identify any candidate variants.

### Unique candidate variants

As a final step, we considered a more heterogenous model in which recurrence in our relatively small cohort may be unlikely. We therefore focused on all predicted pathogenic variants. First, we compared all identified rare *de novo* and/or pathogenic variants to a gene list of previously published candidate genes (Supplementary Table 7). We did not identify any *de novo* or pathogenic variant in a previously published candidate gene (Supplementary Table 8).

Second, we performed an in-silico functional evaluation of all individual variants which were predicted to have a deleterious effect, i.e. high impact consequence, or moderate impact consequence with a CADD_Phred ≥ 30 (Supplementary Table 9). Based on the combination of current available knowledge about the gene function, and lack of overlap between patients and pattern of inheritance, none of the variants were considered as a true candidate.

## Discussion

We present a comprehensive analysis of five patients clinically suspected of having ROHHAD(-NET), summarizing all relevant clinical information and performing an extensive genomic analysis of constitutional aberrations to explore a potential genetic cause of this condition. Using WGS and OGM, we analyzed germline SNVs, InDels and SVs in both coding and noncoding genomic regions. Various models of inheritance were considered, including a *de novo* model, an inherited autosomal dominant, and an autosomal recessive model. Despite our extensive approach using advanced techniques, we did not identify a probable monogenetic cause for ROHHAD(-NET) in the (non)coding regions of the genome.

Based on all available data, including those in the current study, we consider a monogenetic cause for ROHHAD-NET less likely. Several previous genetic studies focusing on the coding region of the genome also failed to find a genetic cause [2, 4–14]. Furthermore, Grossi et al. performed patient-parent germline WGS for two patients suspected for ROHHAD-NET, but did not identify a plausible candidate gene either [14]. To increase chances to identify potential hidden germline aberrations, we were able to apply OGM in two of the patients in our cohort. This technique, similar to long-read sequencing techniques, can overcome limitations of short-read WGS to detect aberrations in e.g. homologous or repetitive regions [25] and, therefore, allow for a much more accurate mapping and variant calling in such regions. Although no candidate gene or locus was identified in these two patients, it may be worthwhile to apply such techniques in larger cohorts of patients suspected for ROHHAD-NET. Larger cohorts may also enable to investigate a more heterogeneous spectrum of monogenetic aberrations or multigenic scenarios of inheritance, as well as the possibility of an epigenetic cause.

A paraneoplastic autoimmune etiology has been proposed and studied as a potential cause for hypothalamic dysfunction in patients with ROHHAD(-NET) [3, 26–30]. Paraneoplastic syndromes are defined as clinical syndromes that are secondary to tumor secretion of functional peptides and hormones or related immune cross-reactivity with normal tissues of the host [31, 32]. This mechanism can explain the sudden onset of symptoms in a previously healthy child with normal development. A rare example of a paraneoplastic syndrome in children with neuroblastoma is opsoclonus-myoclonus syndrome (OMS) [33]. OMS is a neuroinflammatory disorder resulting from a cross-reactive autoimmune respons to both the neuroblastoma and neural cells in the cerebellum and brain stem. Approximately 50% of the patients with OMS are diagnosed with a neuroblastoma, although only 2–3% of the children with a neuroblastoma develop OMS [32, 34, 35]. Similar to patients with OMS, in approximately half of the patients suspected of having ROHHAD(-NET) a neuroendocrine tumor is detected. This relatively low percentage can likely be explained by the fact that neuroendocrine tumors, particularly benign ganglioneuromas, are often asymptomatic or may even have regressed spontaneously by the time ROHHAD(-NET) is diagnosed [36, 37]. There is often a diagnostic delay in children with ROHHAD-NET, due to the rarity of the disease, the exclusion of other potential diagnoses and a need for presentation of more symptoms that match the acronym. Although tumor resection has not been shown to improve symptoms in patients with ROHHAD-NET, this intervention may be considered because of the possible compressive local effect of the tumor and its potential for malignant transformation [38].

In contrast to the well-established understanding of the etiology of OMS, evidence supporting an autoimmune response targeting the hypothalamus in patients with ROHHAD(-NET) is limited [33]. In 2014, Sartori et al. identified oligoclonal bands in the cerebrospinal fluid (CSF) of two patients with ROHHAD(-NET), suggesting the presence of antibody proteins [26]. In one patient, the oligoclonal bands were absent one year after treatment with intravenous immunoglobins. In 2019, Giacomozzi et al. detected anti-pituitary and anti-hypothalamic autoantibodies in a patient with ROHHAD postmortem by indirect immunofluorescence analysis on the CSF [12]. More recently, ZSCAN1 autoantibodies were identified in CSF of 7/9 patients with ROHHAD(-NET), which were absent in a reference cohort of 125 individuals [28]. ZSCAN1 was expressed in the hypothalamus and by the neuroendocrine tumor of one patient. In addition, anti-ZSCAN1 auto-antibodies were present in serum of patients with ROHHAD who did not develop a tumor and absent in control samples in two additional studies [39, 40]. Although little is known about the ZSCAN1 protein, it is an intracellular protein belonging to a subfamily of C2H2 zinc finger transcription factors. These proteins contain an N-terminal SCAN domain and are expressed in the human nervous system and endocrine tissues.

Immunosuppressive agents have been used to manage symptoms of ROHHAD(-NET) [28–30, 41, 42]. Treatment with IVIG, rituximab, and/or high doses of cyclophosphamide has resulted in improvement of symptoms in individual cases. This improvement was often partial, transient, and with limited reported follow-up time. In our cohort, one patient is being treated with IVIG every three weeks possibly resulting in subtle beneficial effects. However, studies evaluating the efficacy of treatment in larger cohorts have not been performed. Consequently the main stay of treatment in these children is within a multidisciplinary team, supportive for oxygenation, endocrine substitution and consistent support for child and family for lifestyle management to minimize the aspects of hypothalamic damage.

A limitation of our study was the sample availability. We have been able to include DNA from five patients with ROHHAD(-NET) in our study, but parental samples were not available for one of the patients, for which inheritance status could not be established. For OGM, a new blood sample was required to extract the UHMW DNA, which was possible for only two patients. Furthermore, we were able to analyse tumor data for only one patient, since the other patients were not diagnosed with a NET or a tumor sample was not available. Despite these limitations, the available data allowed us to thoroughly analyze all potential modes of inheritance.

**Table 1:** Clinical information for each patient suspected for ROHHAD-NET

Characteristics	Patient 1	Patient 2	Patient 3	Patient 4	Patient 5
Gender	Female	Female	Female	Female	Female
Current age	10 years	11 years	7 years	25 years	3 years
**Pregnancy**	Uncomplicated	Uncomplicated	Uncomplicated	Uncomplicated	Uncomplicated
Gestational age	38 weeks	42 weeks	38 weeks	40 weeks	39 weeks + 4
Birth weight (percentile)	3020 gram (P10-50)	3650 gram (P10-50)	4570 gram (*P* > 97)	3500 gram (P50-90)	2374 gram (*P* < 3)
**Hypothalamic Obesity**	Yes*	Yes	Yes	Yes*	Yes*
Age at onset	3.5 years	3 months	3.5 years	2 years	6 months
**Hypoventilation**	Yes	No	Yes*	Yes	Yes
Age at onset	4 years	-	6 months	10 years	2 years
Ventilatory support	DP, NIV	-	NIV	NPPV	Optiflow
**Hypothalamic -pituitary disorder**	Yes	Yes	Yes	Yes	Yes
TSH deficiency	Yes	Yes*	Yes	Yes	Yes
Hypocortisolism	Yes	Yes	No	No	No
Growth hormone deficiency	Yes	No	No	Yes	No
Hypogonadotrophic hypogonadism	Not yet assessed due to age	No	Not yet assessed due to age	Yes	Not yet assessed due to age***
AVP deficiency **	Yes	Yes	No	No	No
Adipsia	Yes	Yes	No	No	No
Hyperprolactemia	Yes	No	No	Yes	No
**Autonomic disfunction**	Yes	Yes	Yes	Yes	Yes
Thermal instability	Yes	No	Yes	Yes	Yes
Bradycardia	Yes	No	Yes	No	No
Eye movement disorder	Oculomotor apraxia	Nystagmus	No	Yes	No
**Treatment**	Yes	Yes	No	Yes	Yes
Methylprednisolone	Yes	No	No	No	No
IVIG	Yes	No	No	No	No
Dexamphetamine	Yes	No	No	No	No
**Metformin**	No	Yes	No	Yes	Yes
**Neuroendocrine tumor**	Yes	No	No	No	Yes
Tumor type (age diagnosis in years)	Ganglioneuroma (4)	-	-	-	Cerebellar ganglion cell tumor (3)****
Treatment NET	Resection at age 7				Partial resection
**Other symptoms**	Yes	Yes	Yes	Yes	Yes
Neurological symptoms	Delayed motor development Seizures Ataxia	Delayed motor development Right-sided n. facialis paresis N. opticus hypoplasia Ataxia	Delayed motor development	Delayed motor development	Delayed motor development
Diagnostic genetic testing	46 XX *PHOX2B*– SNP array– Ataxia gene panel– Trio WES normal	46 XX *PHOX2B*– PWS -, BWS– Trio WES: homozygous p.(Gln50*) in *CRNKL1* gene (VUS)	46 XX *PHOX2B*– PWS– WES normal	46 XX *PHOX2B*– PWS– WES normal	46 XX PWS-, SNP-array-, MLUPD -, WES normal

DP = Diaphragm pacer; NIV = Noninvasive Ventilation; NPPV = Noninvasive Positive-Pressure Ventilation; IVIG = intravenous immunoglobulins; NET = neuroendocrine tumor; *PHOX2B-* = Targeted sequencing and MLPA negative; PWS- = Prader-Willi syndrome testing negative; BWS- = Beckwith-Wiedemann syndrome testing negative (methylation testing and *CDKN1C* sequencing); SNP-array- = No pathogenic CNV’s identified based on SNP array analysis; MLUPD- = normal multilocus uniparental disomy test results; Trio WES = patient-parent whole exome sequencing. * primary presentation in this patient ** AVP deficiency is formarly known as diabetes insipidus *** suspected of development of central precocious puberty **** Somatic *BRAF* mutation

## Conclusion

In conclusion, by performing a comprehensive genome wide data analysis, we did not identify a probable genetic cause for ROHHAD-NET. Based on our observations, we consider a monogenetic cause for this condition less likely. Nevertheless, a polygenetic or epigenetic alternation has not been investigated and may require a larger cohort of patients. In addition, recent findings, including the detection of autoantibodies in the cerebrospinal fluid and the (transient) positive effect of immunosuppressive therapies, are in favor of an autoimmune hypothesis as a potential underlying mechanism for the signs and symptoms of ROHHAD.

## Supplementary Information

Below is the link to the electronic supplementary material.

Supplementary Material 1

## Data Availability

The dataset supporting the conclusions of this article is included within the article and its additional file. Additional information is available upon request.

## Abbreviations

AVP: Arginine vasopressin
BWS: Beckwith-Wiedemann syndrome
CNV: Copy number variant
CSF: Cerebrospinal fluid
dbVar: NCBI dbVar Curated Common Structural Variants
DGV: Database of Genomic Variants: Structural Variation
DP: Diaphragm pacer
IGV: Integrative Genomics Viewer
InDel: Small insertion/deletion
MLUPD: Multilocus uniparental disomy
MNV: Multi nucleotide variants
NET: Neuroendocrine tumor
NIV: Noninvasive ventilation
NPPV: Noninvasive positive-pressure ventilation
OGM: Optical genome mapping
OMS: Opsoclonus-myoclonus syndrome
PR: Paired read
PWS: Prader-Willi syndrome
ROHHAD: Rapid-onset obesity, hypothalamic dysfunction, hypoventilation, autonomic dysregulation
SNV: Single nucleotide variant
SR: Split read
SV: Structural variant
TSH: Thyroid stimulating hormone
UHMW: Ultra-high molecular weight
VAF: Variant allele frequency
VEP: Variant Effect Predictor
WES: Whole exome sequencing
WGS: Whole genome sequencing
